# Renal protection induced by physical exercise may be mediated by the irisin/AMPK axis in diabetic nephropathy

**DOI:** 10.1038/s41598-022-13054-y

**Published:** 2022-05-31

**Authors:** Guilherme Pedron Formigari, Marcella Neves Dátilo, Beatriz Vareda, Ivan Luiz Padilha Bonfante, Claudia Regina Cavaglieri, Jacqueline M. Lopes de Faria, José B. Lopes de Faria

**Affiliations:** 1 Renal Pathophysiology Laboratory, Investigation On Diabetes Complications, State University of Campinas (UNICAMP), Rua Tessália Vieira de Camargo, 126 – Cidade Universitária, Campinas, SP 13083-887 Brasil; 2 Laboratory of Exercise Physiology, School of Physical Education, State University of Campinas (UNICAMP), Campinas, SP Brazil

**Keywords:** Diseases, Nephrology

## Abstract

In patients with diabetes, it has been suggested that physical exercise may reduce albuminuria and the progression of renal disease. However, the molecular mechanism by which physical exercise protects the kidney in diabetes remains poorly understood. The aim of the present study was to determine the contribution of muscle irisin secretion induced by aerobic physical exercise with the subsequent activation of AMPK for kidney protection under diabetic conditions. Aerobic physical exercise in rats protected the kidney in streptozotocin-induced diabetes. It reduced albuminuria, glomerular hypertrophy, and glomerular expression of collagen IV and fibronectin, as well as markers of kidney inflammation, when compared to sedentary diabetic rats. These effects were associated with elevation in muscle FNDC5/irisin and activity of AMPK in the diabetic kidney. However, the beneficial effects of exercise were lost when the diabetic rats were treated with CycloRGDyK, that in the bone it has been described as an irisin receptor blocker. In cultured human tubular (HK-2) cells, treatment with recombinant irisin counteracted the effect of high glucose in a dose-dependent manner. Irisin, per se, also activated AMPK in HK-2 cells. It is concluded that in diabetes, the renal protective effect of exercise may be mediated by the irisin/AMPK pathway.

## Introduction

Despite considerable advances in the management of diabetic nephropathy, it remains the main cause of end-stage renal failure in most parts of the world^1^. Therefore, new strategies for the treatment and prevention of this serious complication of diabetes mellitus (DM) are urgently needed.

Physical exercise may be beneficial for the prevention and treatment of a wide variety of chronic diseases, including, but not restricted to, cardiovascular disease, diabetes, hypertension, and certain types of cancer^2^. In chronic kidney disease (CKD), it has been suggested that physical exercise may improve renal function, although no definitive study has shown that physical exercise can slow the progression of renal function in CKD patients^3^. In type 1 diabetic subjects, intense physical activity is associated with the prevention of the development of microalbuminuria, as well as a reduction in the progression of nephropathy^4^. In experimental diabetic nephropathy, a nephroprotective effects of aerobic physical exercise has been demonstrated^5^. However, the molecular mechanism behind this protection remains poorly understood.

Irisin is a myokine secreted by skeletal muscle in response to exercise^6^. Importantly, a recent study identified the αV class integrins as the receptor of irisin in bone^7^. It has been suggested that this muscle-derived circulating factor could be the link between physical exercise and organ protection^6^. Indeed, irisin may mediate the effects of physical exercise in rescuing synaptic plasticity and memory in models of Alzheimer’s^8^. In another study, it was found that in the kidney, fibrosis was diminished by irisin, and this effect was mediated by the inhibition of the transforming growth factor (TGF) receptor β type 1^9^. Thus, the authors of that study concluded that irisin mediated crosstalk between the muscle and the kidney to reduce kidney fibrosis^9^. That irisin has nephroprotective action has also been shown in the animal model of acute kidney injury (AKI), namely the ischemia/reperfusion (I/R) model^10,11^. In these reports, it was suggested that irisin protects the kidney by reducing tubular cell death via p53^11^ and increasing UCP2 with consequent reduction in apoptosis^10^. In nondiabetic^12,13^ and diabetic^14^ subjects, cross-sectional studies have shown an association between lower plasma levels of irisin and CKD. However, the observations of these studies do not allow us to establish a causal link between plasma irisin levels and CKD. The possible contribution of irisin to the induction of kidney protection through exercise in diabetes has not yet been investigated.

Activation of AMP kinase (AMPK) is a potential strategy for treating diabetic nephropathy^15^. Irisin may be able to activate AMPK in vitro in myoblasts exposed to high glucose^16^ and in human microvascular endothelial cells treated with bacterial lipopolysaccharides (LPS)^17^. In vivo, it has been demonstrated that irisin stimulates the activity of AMPK in the I/R model of AKI^11^. However, the role of irisin in the activation of kidney AMPK under diabetic conditions remains elusive.

In this study, we sought to determine the contribution of muscle irisin secretion induced by aerobic physical exercise with the subsequent activation of AMPK for kidney protection under diabetic conditions.

## Results

### Aerobic physical exercise protects the kidney in diabetic rats, and it is associated with elevation in muscle FNDC5-irisin and renal AMPK activity

We observed consistent improvement in the performance of the exercised rats from baseline until eight weeks (Supplementary Figures 1C-E). Rats with diabetes induced by STZ displayed a reduction in body weight and hyperglycemia. These parameters were not modified by physical exercise. Systolic and diastolic blood pressure were similar in nondiabetic control rats and in rats with DM. However, physical exercise significantly reduced systolic blood pressure. UACR was higher in diabetic rats than in control rats, and this elevation in albuminuria was prevented by physical exercise (Table 1).

**Table 1:** Physical and metabolic parameters of the experimental groups following eight weeks of aerobic exercise training.

Parameters	CT (n = 13)	DM (n = 13)	DM + Exe (n = 9)
Body weight (g)	556.5 ± 58.5	310.9 ± 49.1^#^	292.0 ± 40.3^#^
Fasting glucose (mg/dL)	77.8 ± 9.8	496.7 ± 67.6^#^	461.0 ± 50.3^#^
Systolic blood pressure (mmHg)	150.6 ± 17.2	161.0 ± 18.2	140.6 ± 18.3*
Diastolic blood pressure (mmHg)	83.0 ± 24.2	100.1 ± 29.3	81.5 ± 26.5
Serum creatinine (mg/dL)	0.79 ± 0.3	0.89 ± 0.3	0.79 ± 0.3
UACR (mg/g)	2.4 ± 0.3	3.3 ± 0.6^#^	2.5 ± 0.7*

Values are means ± SE. UACR, urinary albumin-to-creatine ratio. CT, nondiabetic rats; DM, sedentary diabetic rats; DM + Exe, exercised diabetic rats. ^#^*p* < 0.05 vs. CT; *p < 0.05 vs. DM.

**Figure 1:**
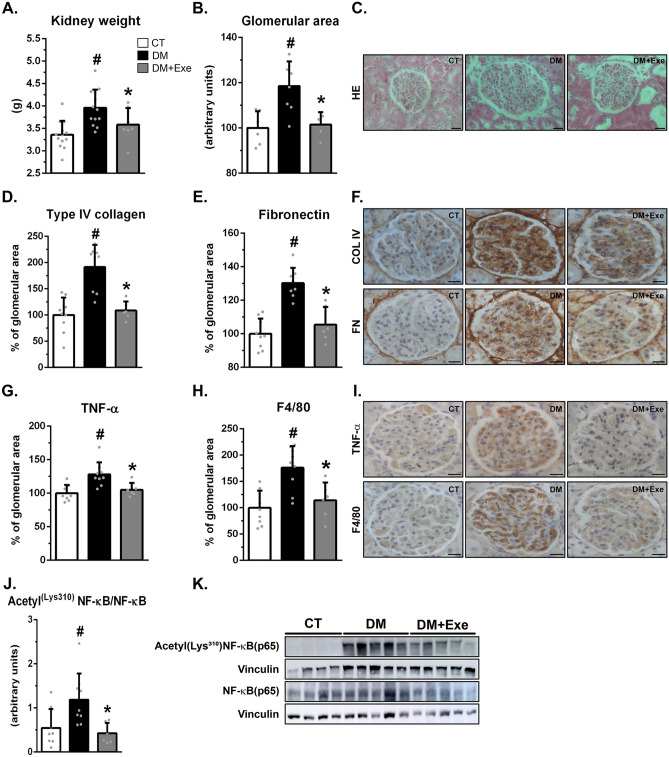
Eight weeks of aerobic physical exercise in diabetic rats prevented markers of diabetic nephropathy. (**A**) Kidney weight. (**B**) Graphical quantitation of the glomerular area. (**C**) Representative photomicrography of renal tuft stained with HE (magnification: × 630). (**D**, **E**, **G**, **H**) Graphical quantitation of immunohistochemistry for type IV collagen (**D**), fibronectin (**E**), TNF-α (**G**), and macrophage infiltration (F4/80, **H**). (**F**, **I**) Photomicrography of glomerular immunohistochemistry for type IV collagen and fibronectin (**F**) and TNF-α and F4/80 (**I**) (magnification: × 630). (**J**, **K**) Western blot analysis of acetyl(Lys^310^)NF-κB(p65) in the renal cortex (**K**), followed by quantitation of acetyl(Lys^310^)NF-κB(p65)/vinculin ratio (**J**). Blots are representative of three independent experiments. Results are means ± SE. CT, nondiabetic; DM, sedentary diabetic; DM + Exe, exercised diabetic. HE, hematoxylin eosin; col IV, collagen IV; FN, fibronectin; F4/80, marker of macrophage infiltration. ^#^*p* < 0.05 vs. CT; *p < 0.05 vs. DM. Scale bars = 20 μm.

**Figure 2:**
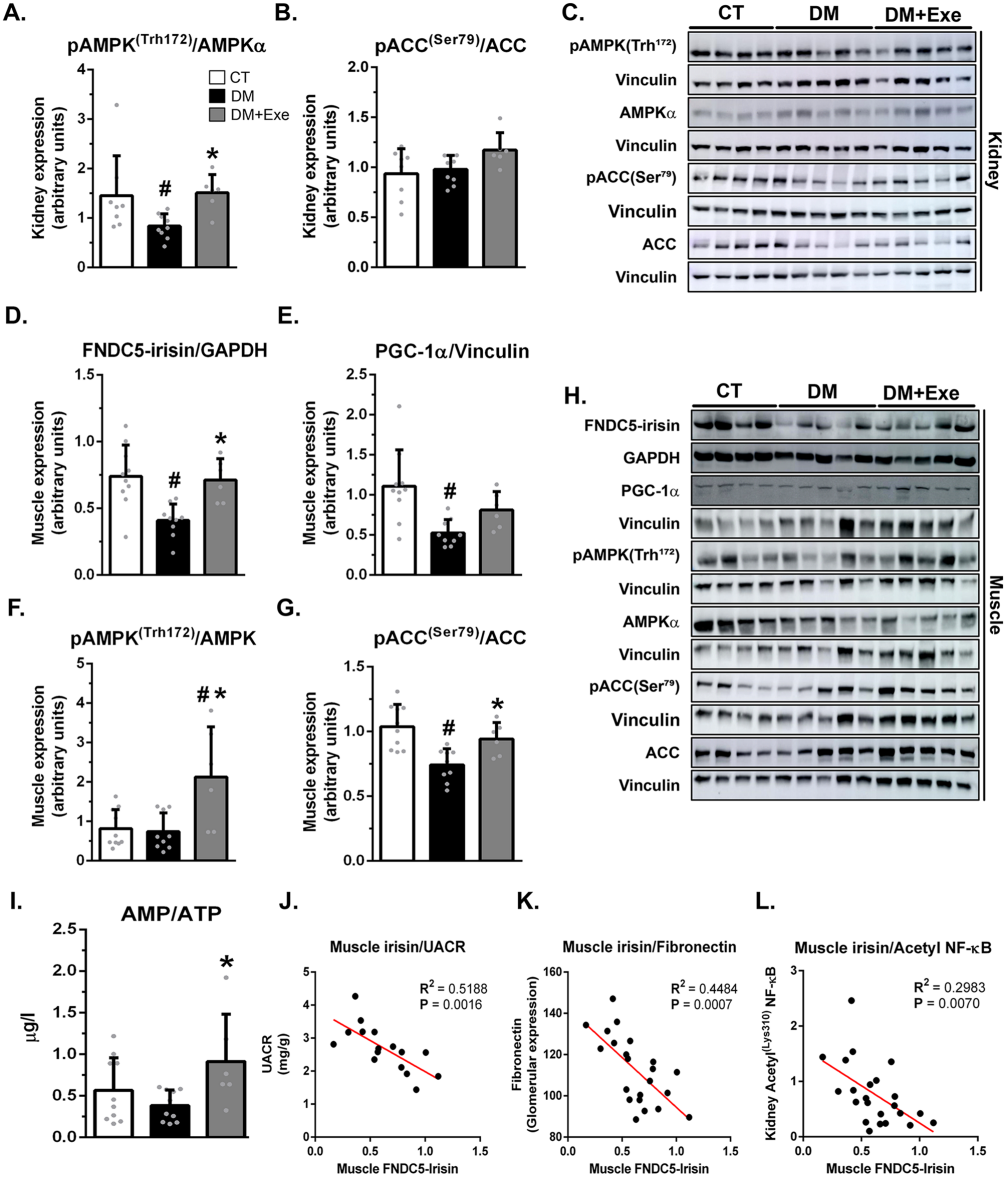
Eight weeks of aerobic physical exercise in diabetic rats was associated with renal and muscle AMPK activation and an elevation in the expression of FNDC5-irisn and PGC1-α in the muscle. (**A**–**C**) Western blot analysis of pAMPK^(Thr172)^, AMPKα, pACC^(Ser79)^, ACC, and vinculin in the renal cortex followed by quantitation of pAMPK^(Thr172)^/vinculin by AMPKα/vinculin ratio (**A**) and pACC^(Ser79)^/vinculin by ACC/vinculin ratio (**B**). (**D**–**H**) Western blot analysis of FNDC5-irisn, GADPH, PGC-1α, vinculin, pAMPK^(Thr172)^, AMPKα, pACC^(Ser79)^, and ACC in the muscle (**H**), followed by quantitation of FNDC5-irisn/GADPH (**D**), PGC-1α/vinculin (**E**), pAMPK^(Thr172)^/vinculin by AMPKα/vinculin (**F**), and pACC^(Ser79)^/vinculin by ACC/vinculin ratio (**G**). (**I**) Muscle AMP/ATP ratio. (**J**–**L**) Correlations of muscle irisin vs. UACR (**J**) as well as muscle irisin vs. glomerular fibronectin (**K**) and renal acetyl NF-κB (**L**). Protein expression levels were normalized to vinculin or GAPDH. Blots are representative of three independent experiments. Results are means ± SE. CT, nondiabetic; DM, sedentary diabetic; DM + Exe, exercised diabetic. UACR, urinary albumin-to-creatine ratio. ACC, acetyl-CoA carboxylase. ^#^*p* < 0.05 vs. CT; *p < 0.05 vs. DM.

Physical exercise reduced classical renal morphological abnormalities associated with experimental DM, such as in kidney weight (Fig. 1A) and in the glomerular area (Fig. 1B, C). It also reduced markers of glomerular fibrosis (the expression of collagen IV (Fig. 1D, F) and fibronectin (Fig. 1E, F)) and markers of kidney inflammation. The latter includes the expression of TNF alpha (Fig. 1G, I), macrophage infiltration (Fig. 1H, I) as assessed by glomerular expression of F4/80 (a glycoprotein expressed by murine macrophages), and the activation of NFKB (Fig. 1J, K) as assessed by its acetylation on lysine 310. Physical exercise also increased the activity of renal AMPK, as assessed by the phosphorylation of AMPK on threonine 175 (Fig. 2A, C) and the elevation of the phosphorylation on serine 79 of the AMPK downstream protein ACC (Fig. 2B, C). However, the elevation in the expression of kidney pACC just failed to reach conventional statistical significance (*p* = 0.069).

These effects of physical exercise in the diabetic kidney were associated with elevation of skeletal muscle expression of FNDC5-irisin (Fig. 2D, H) and its upstream protein, PGC-1 alpha (Fig. 2E, H), the activity of AMPK (Fig. 2F–H), and the AMP/ATP ratio (Fig. 2I). Additionally, we observed a significant correlation between the expression of muscle FNDC5-irisin and albuminuria (Fig. 2J), on the one hand, and the expression of muscle FNDC5-irisin and the glomerular expression of fibronectin (Fig. 2K) and the acetylation of NFKB (Fig. 2L), on the other.

### Blocking the irisin receptor abolished the kidney protection induced by aerobic physical exercise in diabetic rats

**Figure 3:**
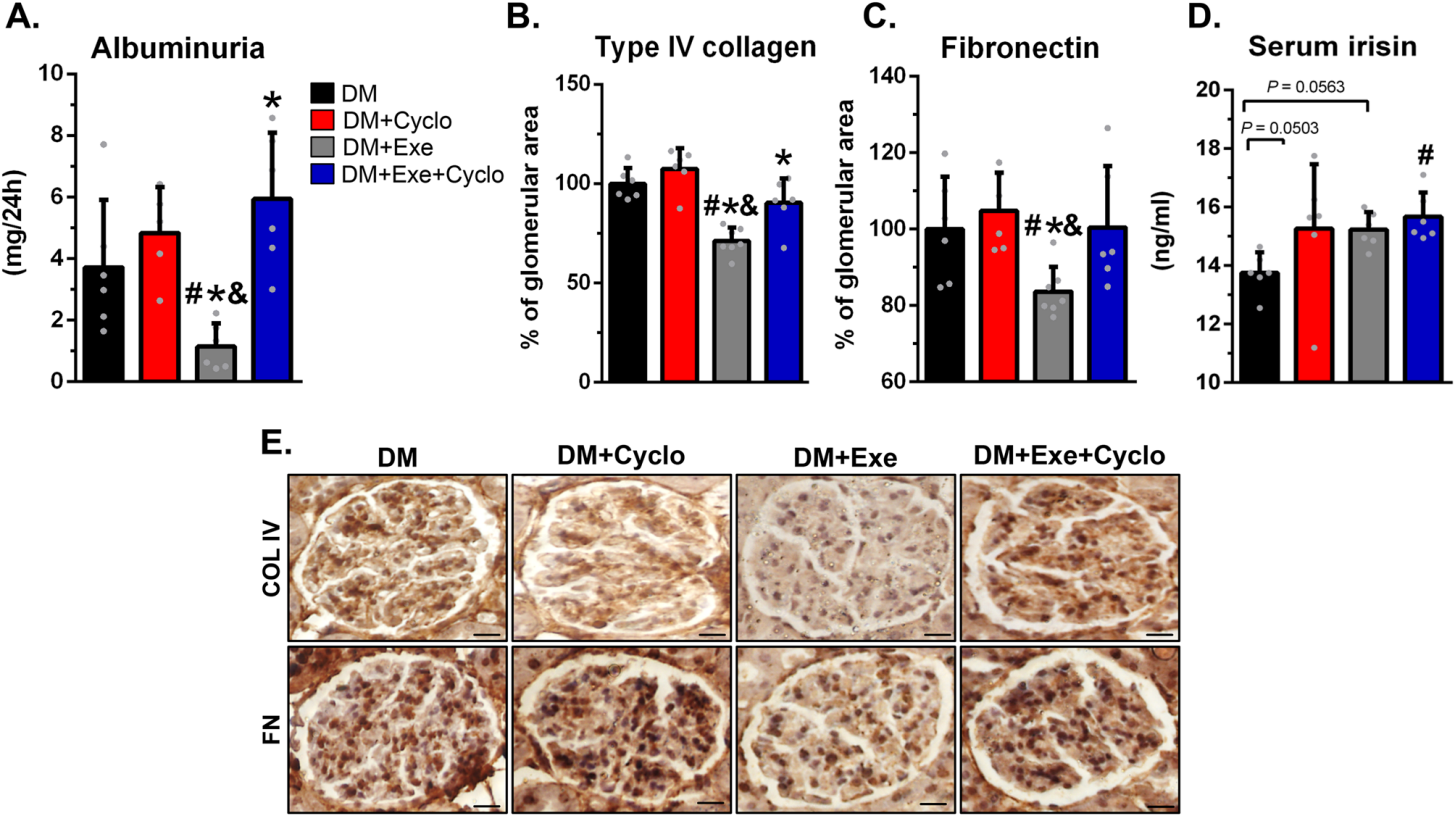
Treatment with an irisin blocker abolished the beneficial effects of aerobic physical exercise in diabetic rats. (**A**) Albuminuria. (**E**) Photomicrography of glomerular immunohistochemistry for type IV collagen and fibronectin (magnification: × 630). (**B**, **C**) Graphical quantitation of immunohistochemistry for type IV collagen and fibronectin. (**D**) Quantitation of serum irisin levels. Results are means ± SE. DM, sedentary diabetic; DM + Cyclo, sedentary diabetic treated intraperitoneally with 1 mg/kg of αV integrin receptor inhibitor (CycloRGDyK); DM + Exe, exercised diabetic rats; DM + Exe + Cyclo, exercised diabetic treated intraperitoneally with 1 mg/kg of CycloRGDyK. ^#^*p* < 0.05 vs. DM, *p < 0.05 vs. DM + Cyclo, and ^&^*p* < 0.05 vs. DM + Exe + Cylo. Scale bars = 20 μm.

To investigate whether the elevation of muscle FNDC5-irisin translates into elevation in serum irisin and whether it participates in the protection of the kidney promoted by physical exercise, we treated the exercised diabetic rats with the recently identified irisin receptor (αV class integrin)^7^ inhibitor (CycloRGDyK), and compared their results with those of untreated exercised diabetic rats. We observed that the administration of CycloRGDyK for eight weeks to exercised or unexercised diabetic rats did not modify their body weight, blood glucose, or systolic and diastolic blood pressure (Table 2). However, the use of the CycloRGDyK prevented the nephroprotection induced by exercise in diabetic rats. It abolished the reduction in albuminuria (Fig. 3A) and diminished the reduction in the glomerular expressions of collagen IV (Fig. 3B, E) and fibronectin (Fig. 3C, E). In diabetic rats, serum irisin levels were elevated by physical exercise (*p* = 0.056) and the use of CycloRGDyK (*p* = 0.050). However, these alterations just failed to reach conventional statistical significance (*p* < 0.05). The concomitance of exercise and treatment with CycloRGDyK in diabetic rats led to a significant elevation in serum irisin levels (Fig. 3D). These findings suggest that irisin may represent the link between the effects of physical exercise in the muscles and the subsequent kidney protection they provide in diabetes.

**Table 2:** Physical and metabolic parameters of experimental groups following eight weeks of aerobic exercise training and treatment with CycloRGDyK.

	DM (n = 8)	DM + CycloRGDyK (n = 7)	DM + Exe (n = 8)	DM + Exe + Cyclo (n = 7)
Body weight (g)	272.4 ± 35.6	278.3 ± 33.1	287.1 ± 31.1	273.6 ± 26.3
**Fasting glucose (mg/dL)**				
After 4 weeks (mg/dL)	506.3 ± 50.0	460.4 ± 37.4	483.8 ± 59.4	523.3 ± 62.3
After 8 weeks (mg/dL)	447.1 ± 31.1	496.0 ± 60.5	437.3 ± 34.8	449.7 ± 72.3
Systolic blood pressure (mmHg)	148.5 ± 12.9	163.9 ± 14.3	152.7 ± 24.7	155.4 ± 15.3
Diastolic blood pressure (mmHg)	77.1 ± 26.0	97.6 ± 34.0	74.2 ± 24.4	107.3 ± 22.8

Results are means ± SE. CT, nondiabetic; DM, sedentary diabetic; DM + Exe, exercised diabetic.

### Irisin prevents the effects of high glucose-induced extracellular matrix accumulation in renal tubular cells in a dose-dependent manner, and it is associated with the activation of AMPK

**Figure 4:**
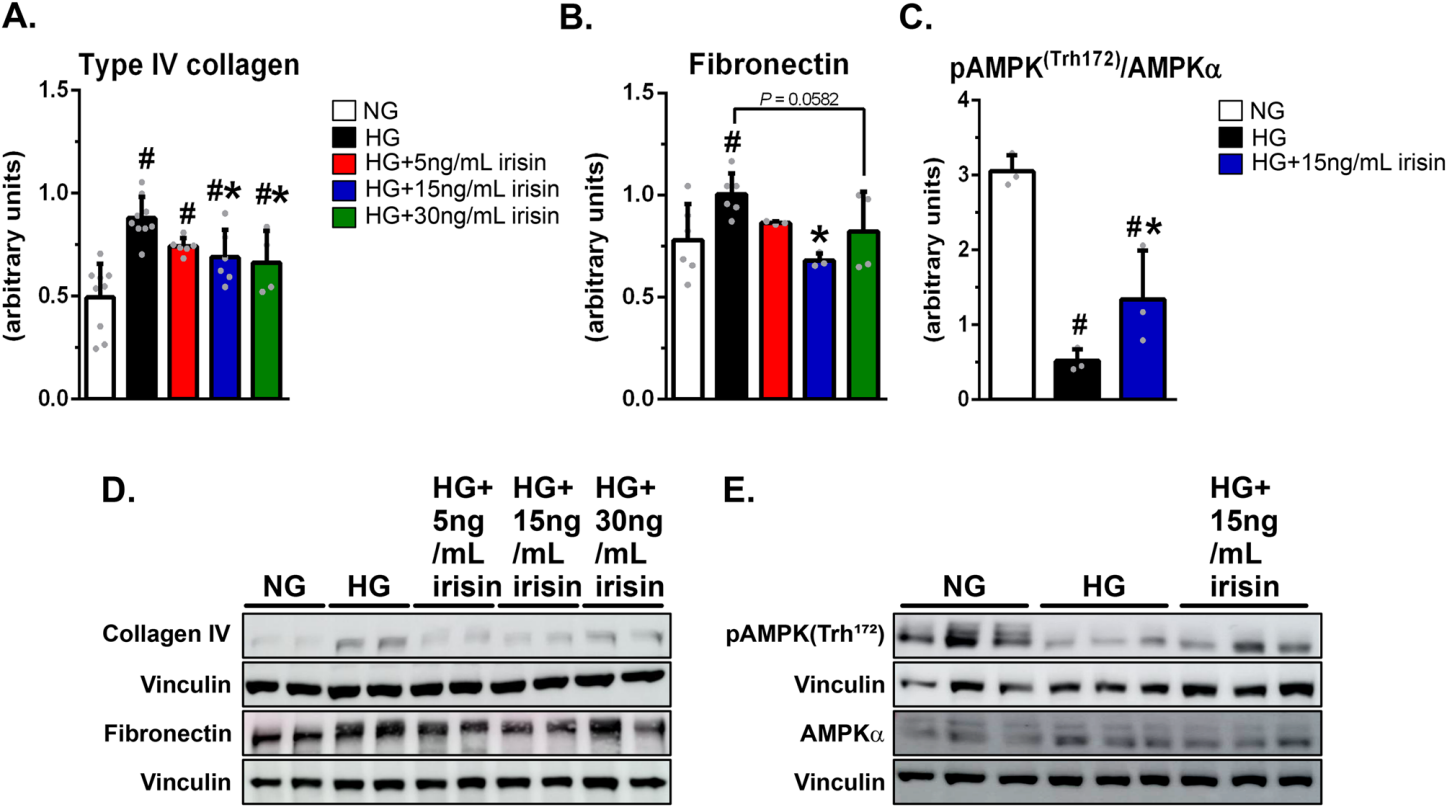
Irisin treatment reduces extracellular matrix accumulation and activates AMPK in HK-2 cells exposed to high glucose. (**D**, **E**) Western blot analysis of type IV collagen, fibronectin, and vinculin (**D**) and pAMPK^(Thr172)^, AMPKα (**E**) in HK-2 cells, followed by quantitation of collagen IV/vinculin (**A**), fibronectin/vinculin (**B**), and pAMPK^(Thr172)^/vinculin by AMPKα/vinculin ratio (**C**). Protein expression levels were normalized to vinculin. Blots are representative of three independent experiments. Results are means ± SE. NG, normal glucose (5.6 mmol/L); HG, high glucose (30 mmol/L); HG + 5 ng/mL, HG plus 5 ng/mL of recombinant irisin; HG + 15 ng/mL irisin, HG plus 15 ng/mL of recombinant irisin; HG + 30 ng/mL irisin, HG plus 30 ng/mL of recombinant irisin. ^#^*p* < 0.05 vs NG, *p < 0.05 vs HG.

Next, we investigated whether irisin per se could ameliorate some classical alterations observed in an in vitro model of diabetic nephropathy. To this end, we tested the effect of treatment with recombinant irisin on the accumulation of extracellular matrix in HK-2 cells exposed to HG (30 mM), mimicking the diabetic milieu. Treatment of HK2 cells with HG promoted elevated expression of collagen IV and fibronectin. These abnormalities were prevented by recombinant irisin in a dose-dependent manner (Fig. 4A, B, D). Fifteen ng/ml of irisin, a value very close to that obtained in our exercised diabetic rats (Fig. 3D), also increased AMPK activity, which had been diminished by treatment with HG in HK-2 cells (Fig. 4C, E). These in vitro findings demonstrate that irisin can prevent the effects of HG in terms of elevated collagen IV and fibronectin in cultured human tubular cells. In addition, they show that the effects of irisin are associated with the activation of AMPK.

### Serum from diabetic subjects submitted to aerobic physical exercise prevented the elevation of extracellular matrix accumulation induced by high glucose in HK-2 cells

**Figure 5:**
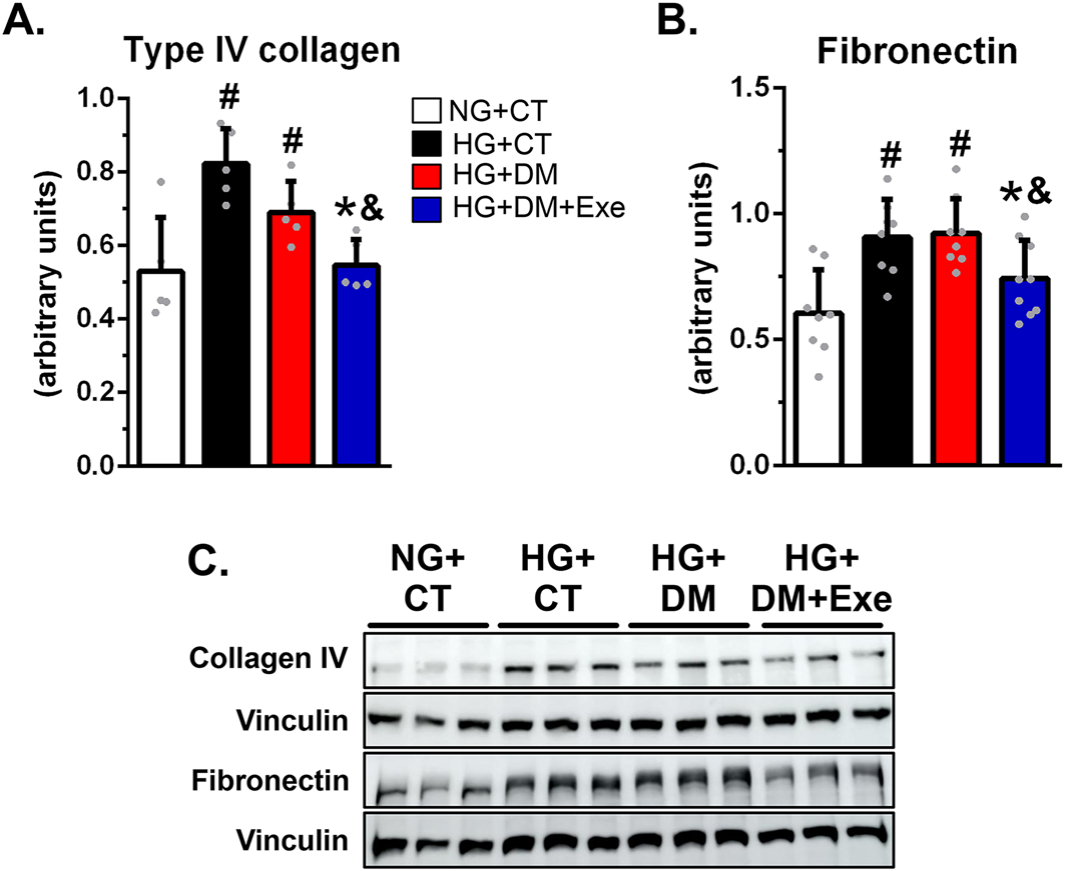
Sera from diabetic exercised individuals and not from sedentary diabetics prevented extracellular matrix accumulation in HK-2 cells exposed to high glucose. (**A**–**C**) Western blot analysis of type IV collagen, fibronectin, and vinculin (**C**) in HK-2 cells, followed by quantitation of collagen IV/vinculin (**A**), and fibronectin/vinculin (**B**). Protein expression levels were normalized to vinculin. Blots are representative of three independent experiments. Results are means ± SE. NG + CT, normal glucose (5.6 mmol/L) plus 4% of serum from non-diabetic patients; HG + CT, high glucose (30 mmol/L glucose) plus 4% of serum from non-diabetic patients; HG + DM, high glucose (30 mmol/L glucose) plus 4% of serum from sedentary diabetic patients; HG + DM + Exe, high glucose (30 mmol/L glucose) plus 4% of serum from diabetic patients submitted to exercise training. ^#^*p* < 0.05 vs NG + CT, *p < 0.05 vs HG + CT, and ^&^*p* < 0.05 vs HG + DM.

Finally, we investigated whether our experimental observation that irisin might mediate the effects of exercise on kidney protection in diabetes could garner any support in a clinical setting. For this, we tested the effects of serum obtained from human diabetic subjects submitted to an aerobic physical exercise program (Supplementary Table 1) in reducing the expression of components of extracellular matrix proteins in HK-2 cells. The expression of collagen IV and fibronectin was higher in HK-2 cells treated with HG and serum from sedentary normal or diabetic subjects than in cells exposed to normal glucose. However, treatment of cells exposed to HG and serum from exercised diabetic subjects showed a significant reduction in the expression of collagen IV and fibronectin (Fig. 5).

## Discussion

**Figure 6:**
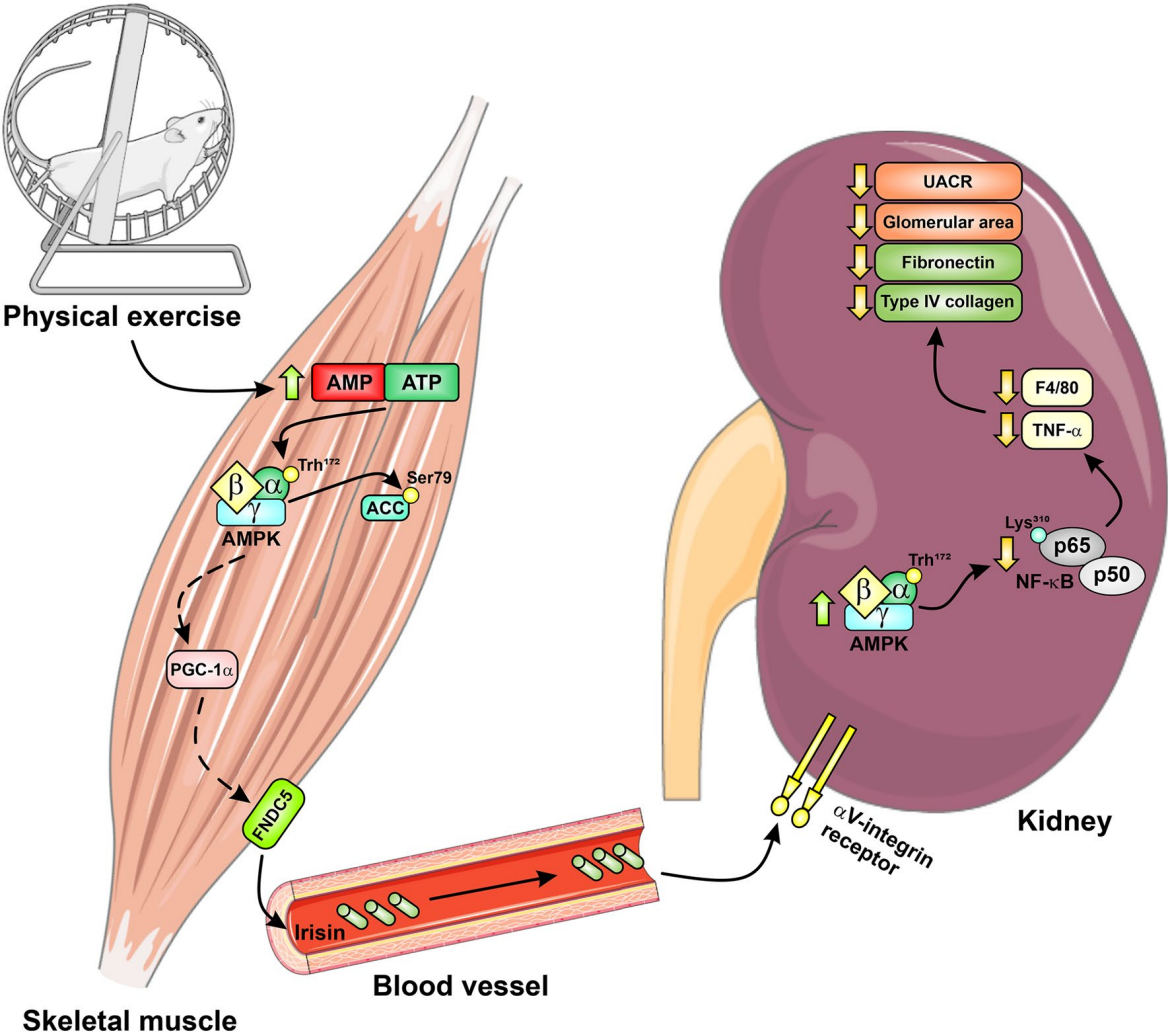
Schematic representation of the mechanism by which irisin/AMPK may mediate the role of physical exercise in inducing renal protection in diabetes. Interrupted arrows linking muscle AMPK, PGC1 and FNDC-5 indicate that the pathway requires further investigation.

Here, we demonstrated that in experimental diabetes, a program of aerobic physical exercise confers nephroprotection and is associated with elevation in muscle and serum irisin, concomitant with elevation in kidney AMPK activity. Furthermore, our in vivo observation that blocking the irisin receptor abolished the protective effects of exercise in the diabetic kidney suggests that irisin might play a crucial role in nephroprotection. This statement is corroborated by the observation of a significant inverse correlation between the muscle expression of FNDC5-irisin and albuminuria, on the one hand, and between FNDC5-irisin and the glomerular expression of fibronectin and NFkB, on the other (Fig. 2J–L). In vitro, recombinant irisin, per se, prevented the effects of HG on ECM accumulation in HK-2 cells in a dose-dependent manner. These effects of irisin were accompanied by the activation of AMPK. Finally, serum from exercised diabetic subjects, but not from sedentary diabetic individuals, could prevent the HG effect of elevating ECM components in HK-2 cells. This result allows us to suggest, to the best of our knowledge for the first time, that in diabetes, irisin/AMPK axis may mediate the kidney protection induced by physical exercise. In Fig. 6, we summarize a possible sequence of events that may determine kidney protection through physical exercise in diabetes.

In diabetic nephropathy, blood glucose and blood pressure control are important factors in the development and progression of the disease^18^. Thus, it is important to note that in the current study, with diabetic rats, physical exercise did not modify blood glucose levels, but it did reduce systolic blood pressure (BP). Therefore, we cannot exclude the possibility that a reduction in systolic BP contributed to the nephroprotection promoted by exercise. It is unclear whether the elevation of irisin by physical exercise contributed to the reduction in BP, although recent data suggest that irisin may reduce BP. Indeed, the acute administration of irisin to spontaneously hypertensive rats has been shown to reduce BP^19^. Interestingly, in this last study, it was suggested that the reduction of BP requires the activation of AMPK because the use of compound C, an AMPK blocker, abolished the effect of irisin on reducing BP.

Glomerular ECM accumulation is a hallmark of diabetic nephropathy^20^. In the present study, physical exercise in diabetic rats reduced the expression of markers of ECM in the glomerular area. Crucially, the effect of physical exercise on reducing glomerular ECM accumulation was lost when diabetic rats were treated with an irisin blocker, the alfa V integrin receptor blocker^7^. In addition, in an in vitro model of diabetic nephropathy, where HK-2 cells were treated with HG, irisin per se inhibited the elevation of the ECM components increased by HG. This piece of data suggests that irisin may play a role in nephroprotection in diabetes.

As expected, in our study, physical exercise activated skeletal muscle AMPK (Fig. 2F–H). It is likely that this was due to a decrease in ATP levels with consequent elevation in the AMP/ATP ratio (Fig. 2I). This then led to the binding of AMP to the regulatory gamma subunit, allosterically activating AMPK. In our diabetic rats, physical exercise was also associated with an elevation in renal AMPK activity. The mechanism by which physical exercise activates renal AMPK is not apparent. However, we speculate that renal AMPK might have been activated by irisin following its binding to a still unidentified renal receptor. Irisin’s ability to activate AMPK has already been suggested under different conditions^16,17,21^. In addition, we observed that irisin per se was able to activate AMPK in HK-2 cells under diabetic conditions (Fig. 4C, E). Therefore, it is plausible that the activation of kidney AMPK contributes to nephroprotection. However, this hypothesis merits further studies for confirmation.

We have assessed the pACC, a downstream of AMPK, as further evidence of AMPK activation^22^. Because pACC was neither diminished nor elevated in the kidneys of diabetic rats, we cannot claim that pACC is involved in the renal protection induced by exercise.

Although there are good experimental data suggesting that irisin may protect different organs^8,9,21^, the same does not apply to humans. Our observation that serum from exercised diabetic subjects, who had elevated serum irisin compared to sedentary diabetic subjects, was able to counteract the effect of high glucose in HEK-2 cells is an interesting one. However, this must be interpreted with caution. Physical exercise can modulate many proteins in the serum, and we have not proven that the beneficial effect of exercise was indeed due to irisin. The question of whether physical exercise may protect the kidney via irisin in diabetic subjects remains open for further investigation.

In sum, our data suggest that in diabetes, renal protection induced by physical exercise may be mediated by irisin/AMPK axis. Further studies using other models of experimental diabetes, probably with the addition of knockout mice, are needed to confirm this original hypothesis. This hypothesis should also be tested in humans.

## Methods

### Animals and study design

All experiments were conducted in accordance with the National Institutes of Health (NIH) Guidelines for the Care and Use of Experimental Animals, complied with the ARRIVE guidelines and were approved by the Research Ethics Committee of the State University of Campinas (UNICAMP) (protocol numbers: 3856-1 and 5569-1/2020). One hundred male Wistar Hannover rats (HanUnib) were obtained from the Multidisciplinary Center for Biological Research (CEMIB) and were used in this study. The rats were housed in groups of three in plastic cages at 21 °C ± 2 with a 12 h light/dark cycle.

In one experiment, diabetes was induced in eight-week-old male Wistar Hannover rats via a single intravenous injection of streptozotocin (STZ, Sigma-Aldrich®, St. Louis, MO; 60 mg/kg in sodium citrate buffer, pH 4.5) via the tail vein. Control rats (nondiabetic) received only the vehicle (citrate buffer). After 48 h, rats with fasting blood glucose ≥ 270 mg/dL were considered diabetic and eligible to participate in these experiments. Rats were allocated to three groups: control (CT, nondiabetic), sedentary diabetic (DM), and diabetic animals submitted to an exercise training protocol on a treadmill (DM + Exe) for eight weeks (Supplementary Figure 1A).

In another experiment, diabetes was induced in eight-week-old male Wistar Hannover rats, as described above. The diabetic rats were allocated to four groups: sedentary diabetic (DM), sedentary diabetic treated intraperitoneally with 1 mg/kg of αV integrin receptor inhibitor^7^ (CycloRGDyK - S7844, Selleck Chemicals®, Houston, TX, USA) five days a week (DM + CycloRGDyK), diabetic exercised animals (DM + Exe), and diabetic exercised rats treated intraperitoneally with 1 mg/kg of CycloRGDyK five days a week (DM + Exe + CycloRGDyK) for eight weeks (Supplementary Figure 1B). CycloRGDyK is a specific inhibitor for integrin class^23,24^, that is widely used in vivo studies^25,26^.

After eight weeks of diabetes, the rats were euthanized with carbon dioxide (CO2) asphyxiation. Anesthetic procedures were used in full, and all precautions were taken to ensure that the animals did not suffer unduly during and after the experimental procedure. The renal cortex and skeletal muscle (gastrocnemius) were harvested and snap-frozen at − 80 °C for future assays.

### Incremental load test and aerobic physical exercise training protocol

Rats were adapted to the exercise on a motor treadmill (AVS Projetos®, São Carlos, São Paulo, Brazil) for five days at 10 min/day, and the exercise intensity was 3 m/min. After two days, the animals were subjected to an incremental load test. The initial test intensity was 6 m/min at a 0% grade, with increments of 3 m/min, every 3 min, until exhaustion. Exhaustion occurred after the animals had reached the end of the treadmill five times. Exhaustion velocity (EV) was determined at the exhaustion point of the test, and 60% of the EV was then used as the exercise training intensity^27,28^. The aerobic exercise training began after diabetes induction and consisted of four weeks of running on a motor treadmill for five days a week at 60% of the EV. The volume of the exercise was gradually increased (at 15, 30, 45, and 60 min) up until the beginning of the fifth week. After this, the exercise volume of the fourth week (60 min) was used over the next four weeks. To compensate for the performance gain during the aerobic physical exercise training, an incremental load test was performed before beginning the exercise protocol (initial), after four weeks of exercise (4-wk), and at the end of aerobic exercise training (8-wk), adjusting the EV percentage for the eight weeks (Supplementary Figures 1A and B).

### Cell culture and treatments

The human renal proximal tubular cell line (HK-2 cells) was obtained from the ATCC (American Type Culture Collection®, Manassas, VA, USA) and maintained in Dulbecco’s modified Eagle’s medium (DMEM) containing 1 g/L glucose supplemented with 10% fetal bovine serum (FBS), 100 U/mL penicillin, and 100 mg/mL streptomycin at 37 °C in a 5% CO_2_ atmosphere. Recombinant irisin was purchased from Adipogen Life Sciences® (San Diego, CA, USA - AG-40B-0136-C010). The HK-2 cells in passages 8 through 12 were left on 1% of FBS for 24 h and exposed to normal glucose (NG) 5.6 mmol/L, high glucose (HG) 30 mmol/L, and HG plus 5, 15, and 30 ng/mL of recombinant irisin for 48 h. This duration (48 h) was chosen after a preliminary test of the cells response to HG under various parameters (Supplementary Figure 2).

We also investigated the effects of treating HK-2 cells cultured in HG with 4% human serum from nondiabetic control, sedentary diabetic, and diabetic patients submitted to an exercise training program. For this purpose, we used five pools from the nondiabetic control group, five pools from sedentary diabetic patients, and five pools from diabetic patients submitted to exercise training. Each of these pools was prepared with serum from three different patients in each group. The sera from these subjects were provided by one of the authors of this manuscript (CRC). These samples were obtained from individuals enrolled in another study that was approved by the local Institutional Review Board (Research Ethics Committee of Faculty of Medical Sciences (FCM), UNICAMP, 1.597.626, 2.030.070, and 4.505.445). The last addendum to the protocol (4.505.445) allowed for the use of the biological material collected for studies like the current one. All patients signed informed consent before enrollment in the study. All procedures were performed in accordance with the guidelines set forth by the Declaration of Helsinki. The clinical characteristics of these subjects are presented in Supplementary Table 1.

### Systolic and diastolic blood pressure

After eight weeks of aerobic exercise training, systolic and diastolic blood pressure were obtained with an optoelectronic-automated device (BP-2000 Blood Pressure Analysis System, Visitech Systems®, Apex, NC). Each systolic and diastolic blood pressure value was taken as the average of three consecutive measurements obtained after stabilization of the readings.

### Determination of albuminuria

The albumin concentration was determined using a sample of a 24-h urine collection by ELISA (Nephrat II®; Exocell, Philadelphia, PA). Urinary and serum creatinine were assessed by a commercially available enzymatic assay (Labtest Diagnostica SA®, Minas Gerais, Brazil - MS 10009010237). Albuminuria was expressed as the urinary albumin-to-creatine ratio (UACR).

### Serum irisin

Serum irisin concentration level was determined using a commercial ELISA kit (Phoenix Pharmaceuticals®, Burlingame, CA, USA - EK-067-29).

### Glomerular area

The kidneys were fixed with 10% buffered formalin and embedded in paraffin for sectioning. Five-micrometer kidney sections were obtained with a microtome (Leica® Microsystems RM2155) and stained with eosin and hematoxylin (HE staining). For glomerular area quantitation, 35 glomeruli from each rat were obtained using a magnification of X630 on an optical microscope (Leica® - DMLB 100S). The quantitation of the glomerular area was performed using ImageJ Software® (National Institutes of Health, Bethesda, MD).

### Immunohistochemistry

Immunohistochemistry was performed on 5-mm-thick sections mounted on glass slides precoated with 2% silane, as previsouly described^29^. The primary antibodies used were: (anti-rabbit polyclonal type IV collagen 1:50 – abcam 6586 [Cambridge, UK); anti-rabbit polyclonal fibronectin 1:50 – abcam 2413; anti-mouse monoclonal TNF-α 1:25 – Santa Cruz Biotechnology sc-52746 [Santa Cruz, CA]; and anti-mouse monoclonal F4-80 1:50 Bio-Rad MCA497RT [Richmond, CA]) diluted in milk at 1%. After this, 35 glomeruli from each rat were obtained using a magnification of X630 on an optical microscope (Leica® – DMLB 100S). The quantitation of renal densities for type IV collagen, fibronectin, TNF-α, and F4/80 positivity in the glomerular area was performed using ImageJ Software® (National Institutes of Health, Bethesda, MD).

### Western blot analysis

The samples (renal cortex and gastrocnemius muscle) and Western blots were prepared and performed as previously described^29,30^. Protein quantification was determined using the Bradford method^31^. Immunoreactive bands were visualized using the enhanced chemiluminescence method (Super Signal CL-HRP Substrate System, Pierce®, Rockford, IL). The uniformity of protein loading and transfer efficiency were assessed by reprobing the membranes for vinculin (rabbit monoclonal anti-vinculin antibody, 1:1000, Cell Signaling Technology®, Boston, USA) or GAPDH (#5174; 1:1000). The images were captured using a digital photo documentator (ImageQuant LAS 500, GE Healthcare Life Sciences®, Boston, USA) and analyzed quantitatively using the ImageJ software® (National Institutes of Health, USA). Three independent experiments were carried out.

The antibodies used in our experiments are listed in Supplementary Table 2.

### Measurement of muscle AMP and ATP levels

Adenosine triphosphate (ATP) and adenosine monophosphate (AMP) were determined in muscle tissue (gastrocnemius) homogenate using HPLC method, as previous described^32^.

### Statistical analysis

Differences among groups were assessed using one-way ANOVA with pairwise post-test comparisons according to the Fisher method. The results are presented as means ± SE. A Pearson correlation coefficient (Pearson r) was used to estimate the correlation between the muscle expression of irisin and albuminuria, as well as between the muscle expression of irisin and the glomerular expression of fibronectin and kidney acetylation of NF-κB (p65). Calculations were performed using GraphPad Prism® 6 software. *P* values of < 0.05 were considered statistically significant.

## Supplementary Information

Supplementary Information 1.

Supplementary Legends.

Supplementary Information 2.

## Data Availability

The datasets generated during and/or analysed during the current study are available from the corresponding author on reasonable request.
