# Supplementary material for: Renal protection induced by physical exercise may be mediated by the irisin/AMPK axis in diabetic nephropathy
Source: Sci Rep. 2022 May 31;12:9062. doi: 10.1038/s41598-022-13054-y (PMC9156698; doi:10.1038/s41598-022-13054-y)
Supplement: Supplementary file 1 — Supplementary Information 1. [file 41598_2022_13054_MOESM1_ESM.pdf]

Supplementary Figure 1

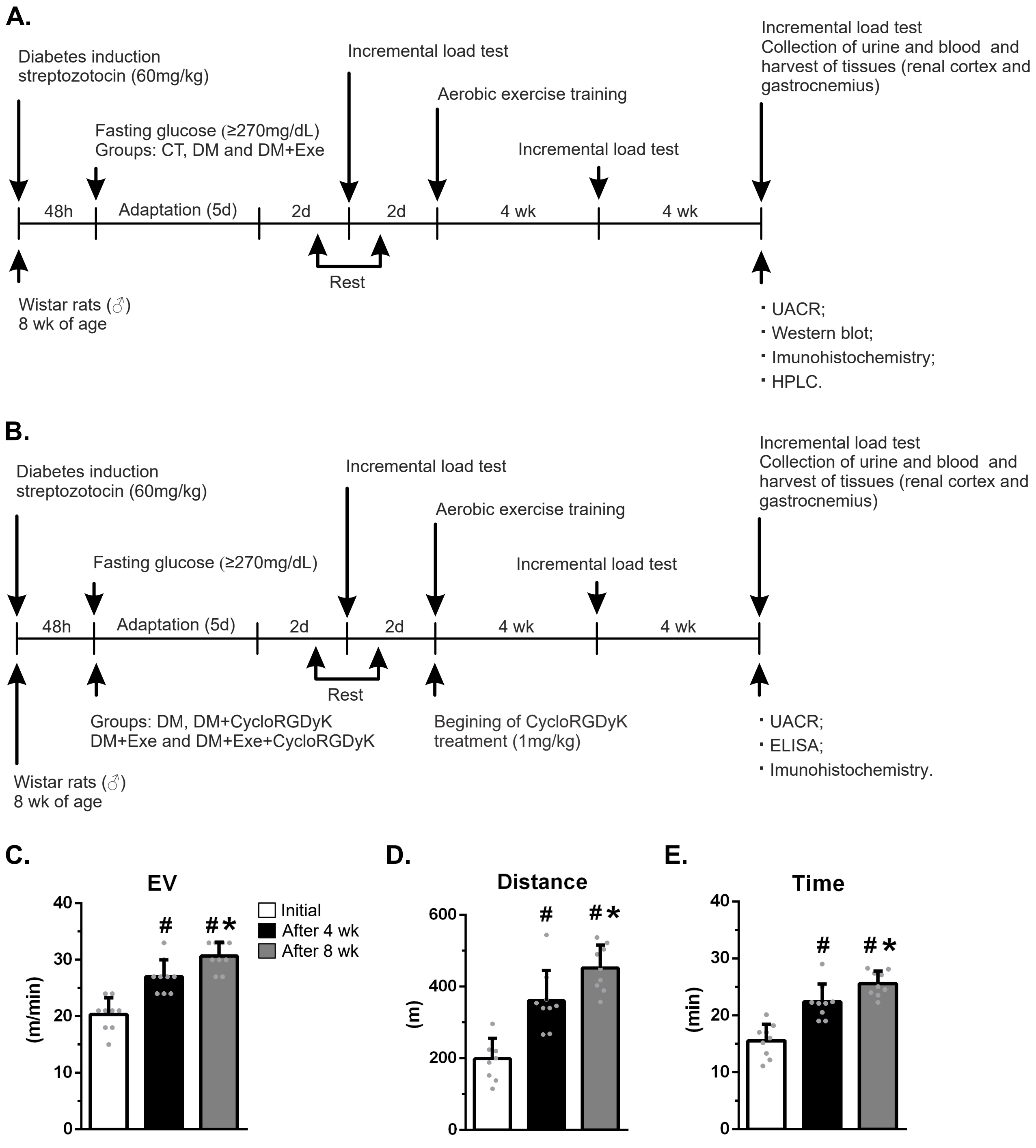

Supplementary Figure 2

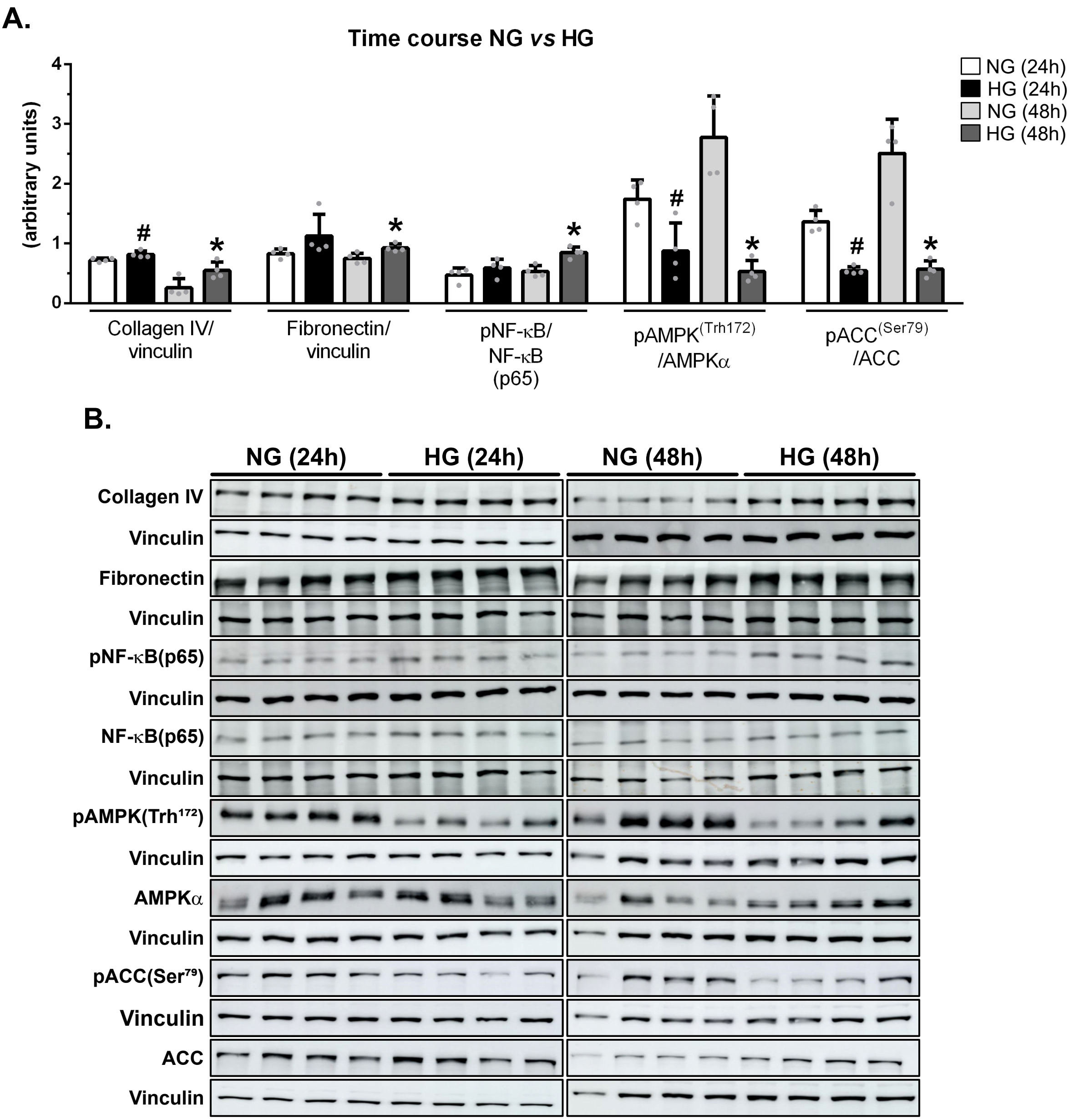

Supplementary Table 1

Parameters	Non diabetic control	Sedentary diabetics	Exercised diabetics
**Gender (male/female)**	6/9	9/6	7/8
**Age (years)**	47.0 ± 4.8	51.7 ± 4.5	50.6 ± 3.9
**BMI (weight/height^2)**	26.8 ± 3.3	30.6 ± 3.1#	29.4 ± 3.6#
**Diabetes diagnosis (years)**	-	4.4 ± 2.0	5.5 ± 2.5
**Fasting glucose (mg/dL)**	84.9 ± 8.7	144.7 ± 40.8#	131.8 ± 30.9#
**Glycated hemoglobin (%)**	NA	7.3 ± 1.3	7.2 ± 1.5
**Systolic blood pressure (mmHg)**	NA	123.3 ± 12.9	113.0 ± 14.6
**Diastolic blood pressure (mmHg)**	NA	79.1 ± 7.4	73.2 ± 9.0
**Serum irisin (μg/mL)**	NA	1.1 ± 0.5	1.7 ± 0.6*

Supplementary Table 2

Antibodies (dilution)	Source	Identifier
**Anti-rabbit phospho-AMPKa Thr172 (1:1000)**	Cell Signaling Technology	Cat. # 2535
**Anti-rabbit AMPKa (1:1000)**	Cell Signaling Technology	Cat. # 5831
**Anti-rabbit phospo-ACC Ser79 (1:1000)**	Cell Signaling Technology	Cat. # 11818
**Anti-rabbit ACC (1:1000)**	Cell Signaling Technology	Cat. # 3676
**Anti-rabbit vinculin (1:1000)**	Cell Signaling Technology	Cat. # 13901
**Anti-rabbit GAPDH (1:1000)**	Cell Signaling Technology	Cat. # 5174
**Anti-rabbit phopho-NF-κB p65 (1:1000)**	Cell Signaling Technology	Cat. # 3033S
**Anti-rabbit PGC1-α (1:250)**	Cell Signaling Technology	Cat. # 2178S
**Anti-rabbit fibronectin (1:100 – WB, 1:50 – IHC)**	Abcam	Cat. # ab2413
**Anti-rabbit type IV collagen (1:50 – IHC)**	Abcam	Cat. # ab6586
**Anti-goat type IV collagen (1:1000)**	SouthernBiotech	Cat. # 1340-01
**Anti-rabbit acetyl - Lys310 - NF-κB p65 (1:500)**	Assay Biotechnology	Cat. # D0018
**Anti-mouse NF-κB p65 (1:500)**	Santa Cruz Biotechnology	Cat. # sc8008
**Anti-mouse TNF-a (1:25 – IHC)**	Santa Cruz Biotechnology	Cat. # sc52746
**Anti-mouse F4-80 (1:50 – IHC)**	Bio-Rad Laboratories	Cat. # MCA497RT
**Anti-rabbit FNDC5/irisin (1:1500)**	Phoenix Pharmaceuticals	Cat. # G-067-16
**Anti-rabbit HRP-linked (1:2000)**	Cell Signaling Technology	Cat. # 7074S
**Anti-goat HRP-linked (1:2000)**	Santa Cruz Biotechnology	Cat. # sc2354
**Anti-mouse HRP-linked (1:2000)**	Thermo Fisher Scientific	Cat. # 31430
**Biotinylated anti-rabbit H+L (1:200)**	Vector Laboratories	Cat. # BA-100
**Biotinylated anti-mouse H+L (1:200)**	Vector Laboratories	Cat. # BA-200
