# Supplementary material for: Renal protection induced by physical exercise may be mediated by the irisin/AMPK axis in diabetic nephropathy
Source: Sci Rep. 2022 May 31;12:9062. doi: 10.1038/s41598-022-13054-y (PMC9156698; doi:10.1038/s41598-022-13054-y)
Supplement: Supplementary file 2 — Supplementary Legends. [file 41598_2022_13054_MOESM2_ESM.docx]

Supplementary Figure 1. A:Experimental protocol to assess the effect of aerobic physical exercise on nephroprotection in diabetic rats. B:Experimental protocol to assess the effect of treatingexercised diabetic rats with an irisin receptor blocker (CycloRGDyK) on nephroprotection induced by physical exercise. C:Incremental load exhaustion velocity (EV) reached in the initial test and after fourand eightweeks of aerobic exercise. D:Incremental load test distance reached in the initial test and after fourand eightweeks of aerobic exercise. E:Incremental load test time reached in the initial test and after fourand eightweeks of aerobic exercise in diabetic rats. CT, nondiabetic; DM, sedentary diabetic; DM + Exe, exercised diabetic; DM + Cyclo, sedentary diabetic treated intraperitoneally with 1mg/kg of αV integrin receptor inhibitor (CycloRGDyK); DM+Exe+Cyclo, exercised diabetic treated intraperitoneally with 1mg/kg of CycloRGDyK. Results are means ± SE. #p< 0.05 vs.initial, *p< 0.05 vs.afterfour weeks.

Supplementary Figure 2. Time course of the effect of high glucose treatment in HK-2 cells. Aand B:Western blot analysis of collagen IV, fibronectin, NF-κB(p65) pAMPK(Thr172), AMPKα, pACC(Ser79), ACC,and vinculin in HK-2 cells treated with high glucose for 24 hoursor 48 hoursfollowed by quantitation of collagen IV/vinculin, fibronectin/vinculin, NF-κB/vinculin, and pAMPK(Thr172)/vinculin by AMPKα/vinculin ratio,and pACC(Ser79)/vinculin by ACC/vinculin ratio.The uniformity of protein loading and transfer efficiency were assessed by reprobing the membranes for vinculin. Blots are representative of three independent experiments. Results are means ± SE. NG, normal glucose (5.6 mmol/L); HG, high glucose(30 mmol/L glucose). #p< 0.05 vs.NG (24h), *p< 0.05 vs.NG (48h).

Supplementary Table 1. Physical and metabolic parameters of diabetic patients. Results are means ± SE. #p< 0.05 vs.nondiabetic control, *p< 0.05 vs.sedentary diabetics.

Supplementary Table 2. List of antibodies used in the experiments.
