# Supplementary material for: Renal protection induced by physical exercise may be mediated by the irisin/AMPK axis in diabetic nephropathy
Source: Sci Rep. 2022 May 31;12:9062. doi: 10.1038/s41598-022-13054-y (PMC9156698; doi:10.1038/s41598-022-13054-y)
Supplement: Supplementary file 3 — Supplementary Information 2. [file 41598_2022_13054_MOESM3_ESM.pdf]

Renal protection induced by physical exercise may be mediated by the irisin/AMPK axis in diabetic nephropathy

Guilherme Pedron Formigari,[1] Marcella Neves Dátilo,[1] Beatriz Vareda[1], Ivan Luiz Padilha Bonfante,[2] Claudia Regina Cavaglieri,[2] Jacqueline M Lopes de Faria,[1] José B Lopes de Faria[1*]

[1]Renal Pathophysiology Laboratory, Investigation on Diabetes Complications, State University of Campinas (UNICAMP), Campinas, SP, Brazil.

[2]Laboratory of Exercise Physiology, School of Physical Education, State University of Campinas (UNICAMP), Campinas, SP, Brazil.

Figure 1K ^{Acetyl}(Lys^{310}) NF-KB(p65)/NF-KB(p65)

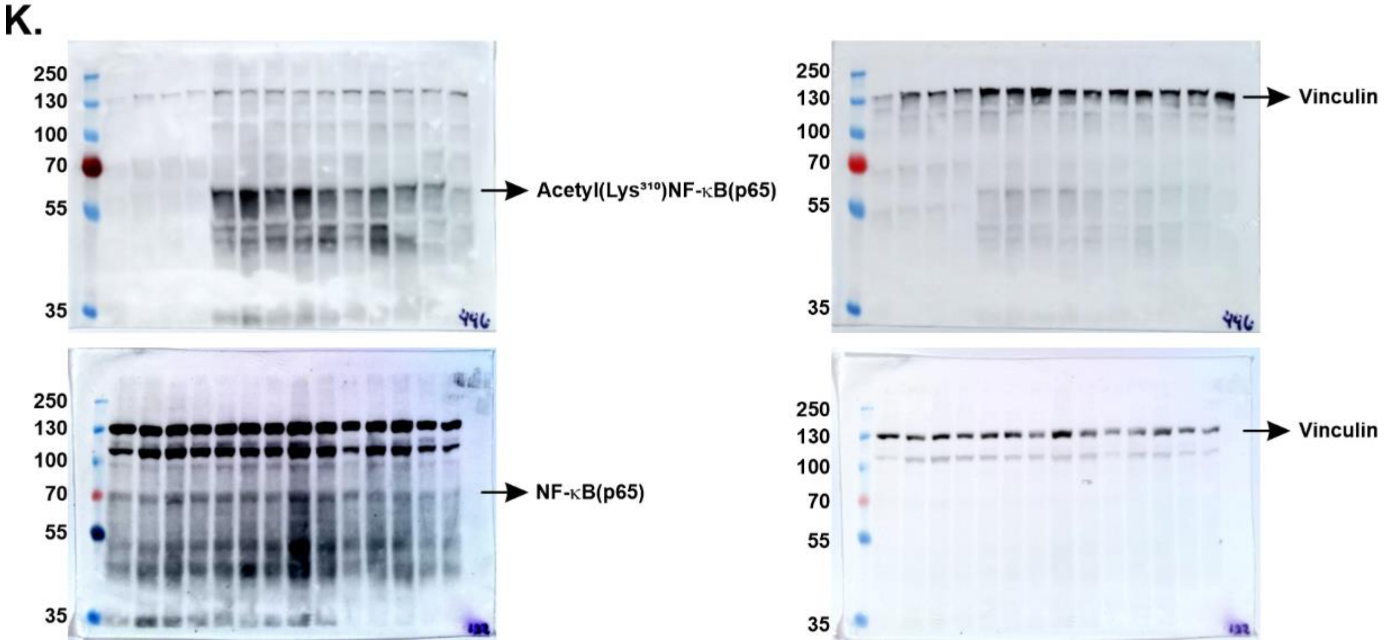

There are full-length gels and blots of ^{Acetyl}(Lys^{310})NF-KB(p65) (top left), its Vinculin (top right), NF-KB(p65) (bottom left), and its Vinculin (bottom right) in Figure 1K. In the four gels, from left to right, the first blots represent the molecular markers, the second set of four blots represents the expression level in the kidneys of control rats (CT), the third set of five blots represents the expression level in the kidneys of diabetic rats (DM), and the fourth set of five blots represents the expression level in the kidneys of diabetic exercised rats (DM + Exe).

Figure 2C pAMPK(Trh^{172})/AMPKα and pACC(Ser^{79})/ACC

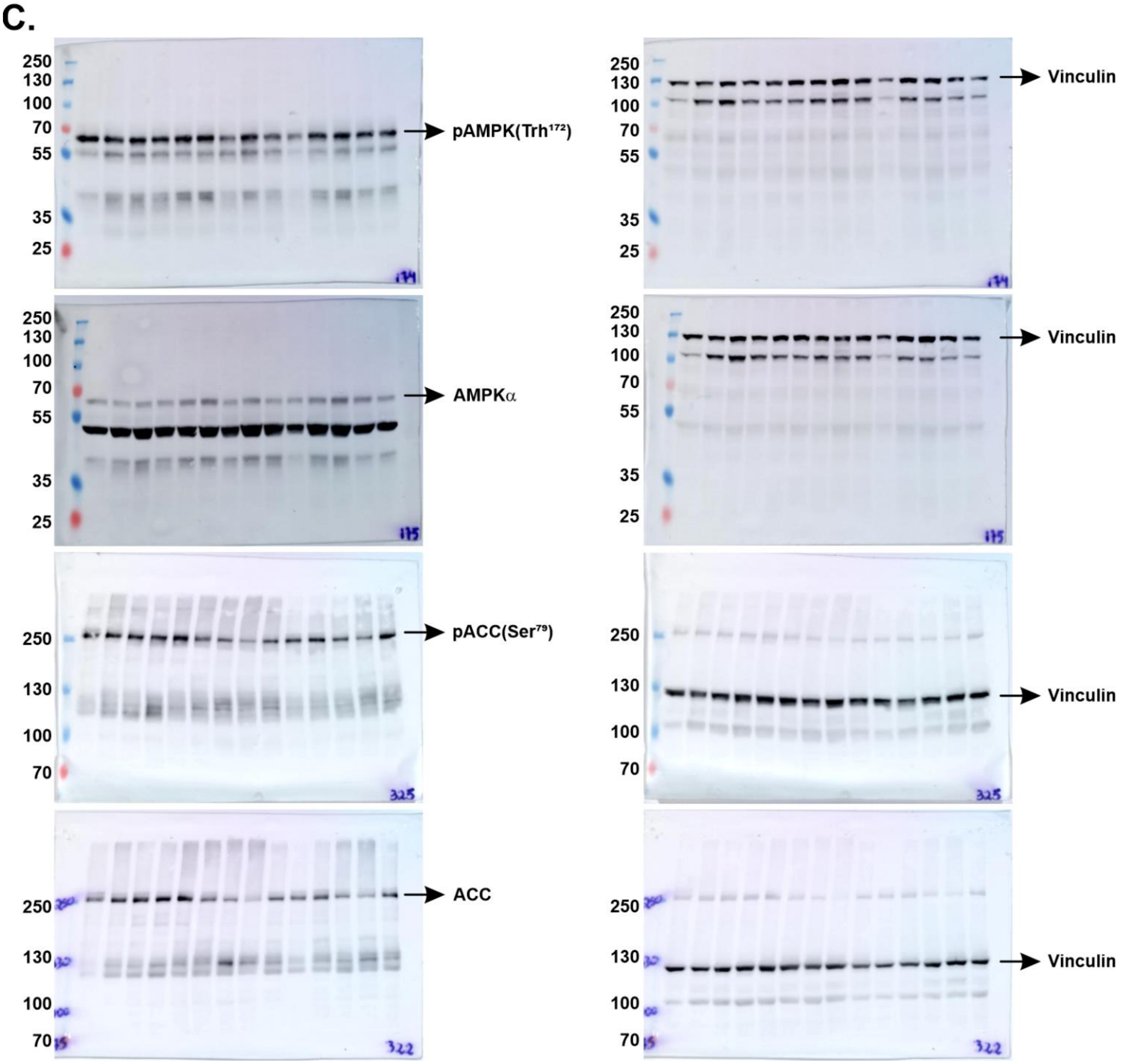

There are the full-length gels and blots of pAMPK(Trh172)/AMPKα and pACC(Ser79)/ACC (left), and its Vinculin (right) in Figure 2C. In the eight gels, from left to right side, the first blots represent the molecular markers, the second set of four blots represent the expression level in the kidney of control rats (CT), the third set of five blots represent the expression level in the kidney of diabetic rats (DM), and the fourth set of

five blots represent the expression level in the kidney of diabetic exercised rats (DM + Exe).

Figure 2H FNDC5-irisin/Vinculin, PGC-1α/Vinculin, pAMPK(Trh[172])/AMPKα and pACC(Ser[79])/ACC

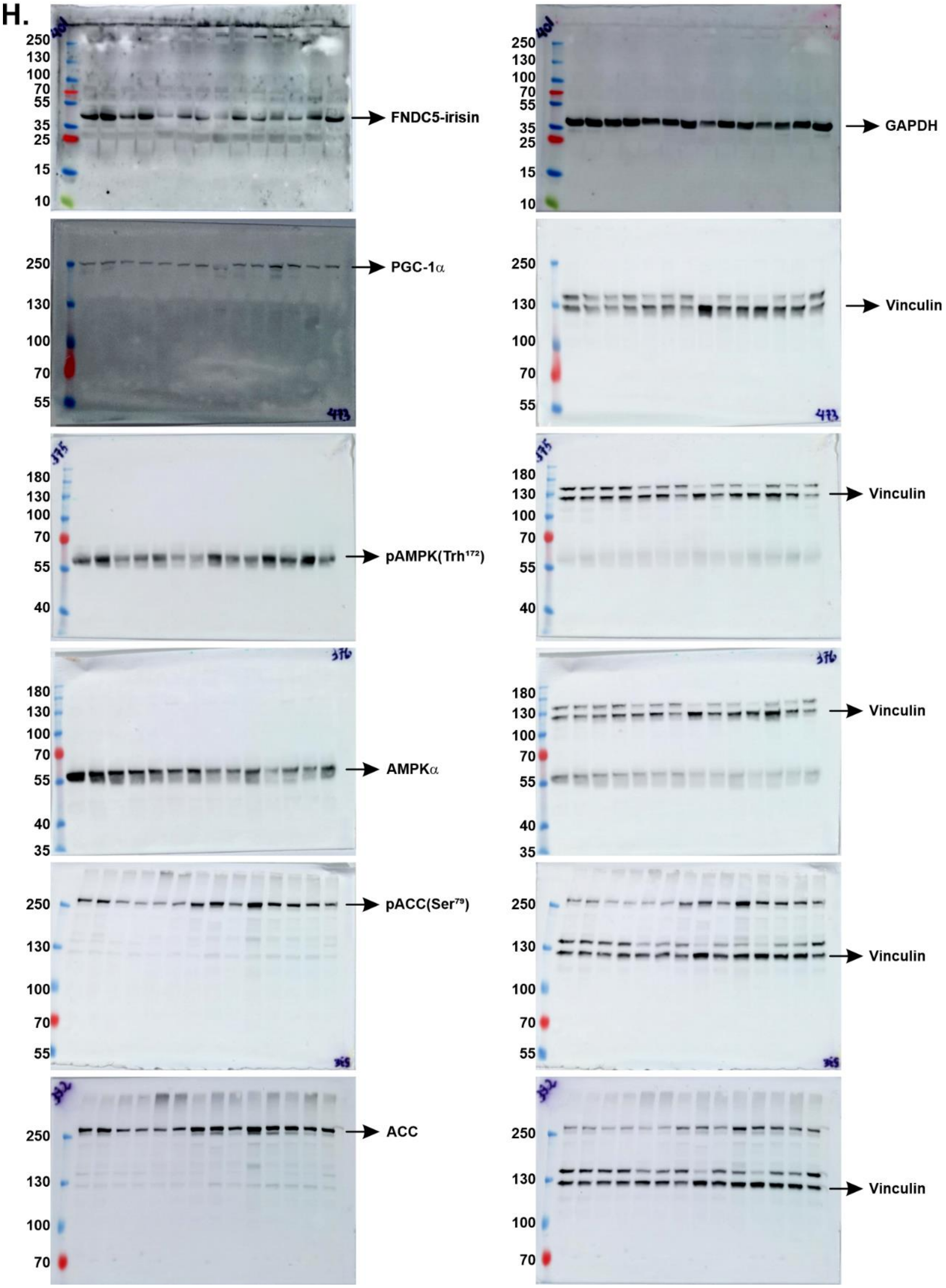

There are the full-length gels and blots of FNDC5-irisin, PGC-1α, pAMPK(Trh^{172})/AMPKα and pACC(Ser79)/ACC (left), and its GAPDH or Vinculin (right), in Figure 2H. In the twelve gels, from left to right side, the first blots represent the molecular markers, the second set of four blots represent the expression level in the skeletal muscle (gastrocnemius) of control rats (CT), the third set of five blots represent the expression level in the skeletal muscle (gastrocnemius) of diabetic rats (DM), and the fourth set of five blots represent the expression level in the skeletal muscle (gastrocnemius) of diabetic exercised rats (DM + Exe).

Figure 4D-E Type IV collagen/Vinculin, Fibronectin/Vinculin and pAMPK(Trh^{172})/AMPKα

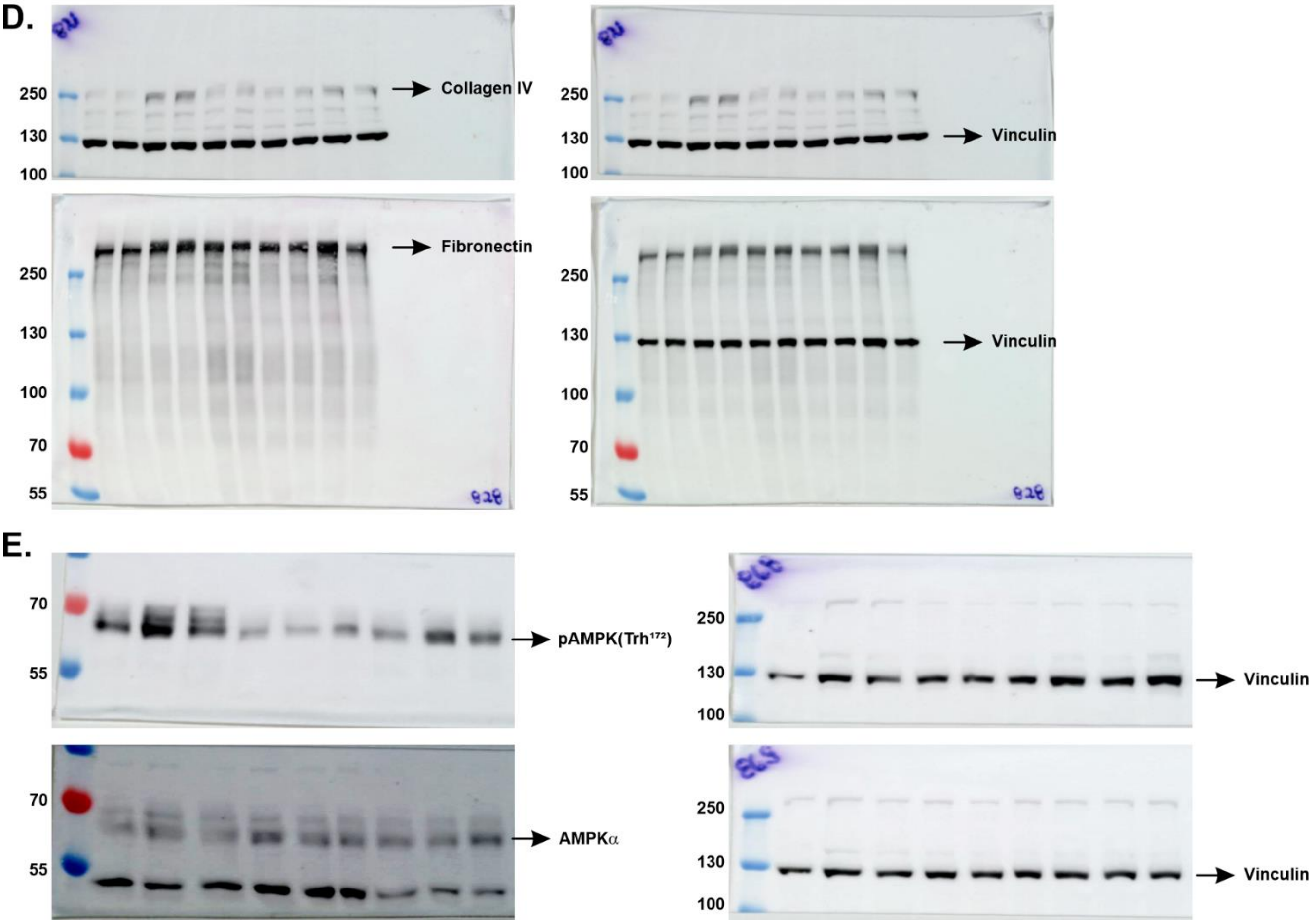

There are the full-length gels and blots of type IV collagen, fibronectin and pAMPK(Trh172)/AMPKα (left), and its Vinculin (right), in Figure 4D-E. In the gels from Figure 4D, from left to right side, the first blots represent the molecular markers, the second set of two blots represent the expression level in HK-2 cell exposed to NG, the third set of two blots represent the expression level in HK-2 cell exposed to HG, the fourth set of two blots represent the expression level in HK-2 cell exposed to HG plus 5 ng/mL of recombinant irisin, the fifth set of two blots represent the expression level in HK-2 cell exposed to HG plus 15 ng/mL of recombinant irisin, and the sixth set of two blots

represent the expression level in HK-2 cell exposed to HG plus 30 ng/mL of recombinant irisin. In the gels from Figure 4E, from left to right side, the first blots represent the molecular markers, the second set of three blots represent the expression level in HK-2 cell exposed to NG, the third set of three blots represent the expression level in HK-2 cell exposed to HG, and the fourth set of three blots represent the expression level in HK-2 cell exposed to HG plus 15 ng/mL of recombinant irisin.

Figure 5C Type IV collagen/Vinculin and Fibronectin/Vinculin

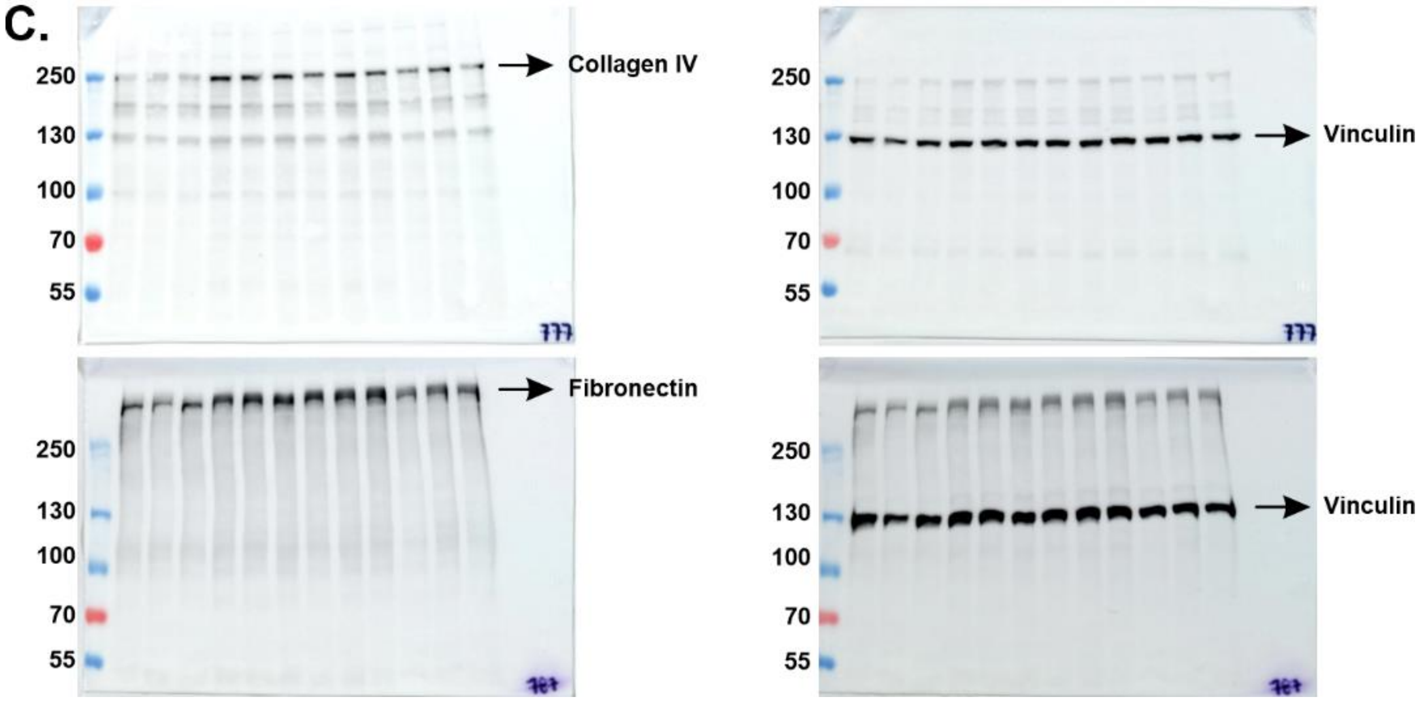

There are the full-length gels and blots of type IV collagen and fibronectin (left), and its Vinculin (right), in Figure 5C. In the four gels, from left to right side, the first blots represent the molecular markers, the second set of three blots represent the expression level in HK-2 cell cultured in NG with 4% human serum from non-diabetic control patients (CT), the third set of three blots represent the expression level in HK-2 cultured in HG with 4% human serum from non-diabetic control patients (CT), the fourth set of three blots represent the expression level in HK-2 cell cultured in HG with 4% human serum from sedentary diabetic patients (DM), the fifth set of three blots represent the expression level in HK-2 cell cultured in HG with 4% human serum diabetic patients submitted to an exercise training (DM+Exe).

Supplementary Figure 2B Type IV collagen/Vinculin, Fibronectin/Vinculin, pNF-κB/NF-κB(p65), pAMPK(Trh[172])/AMPKα and pACC(Ser[79])/ACC

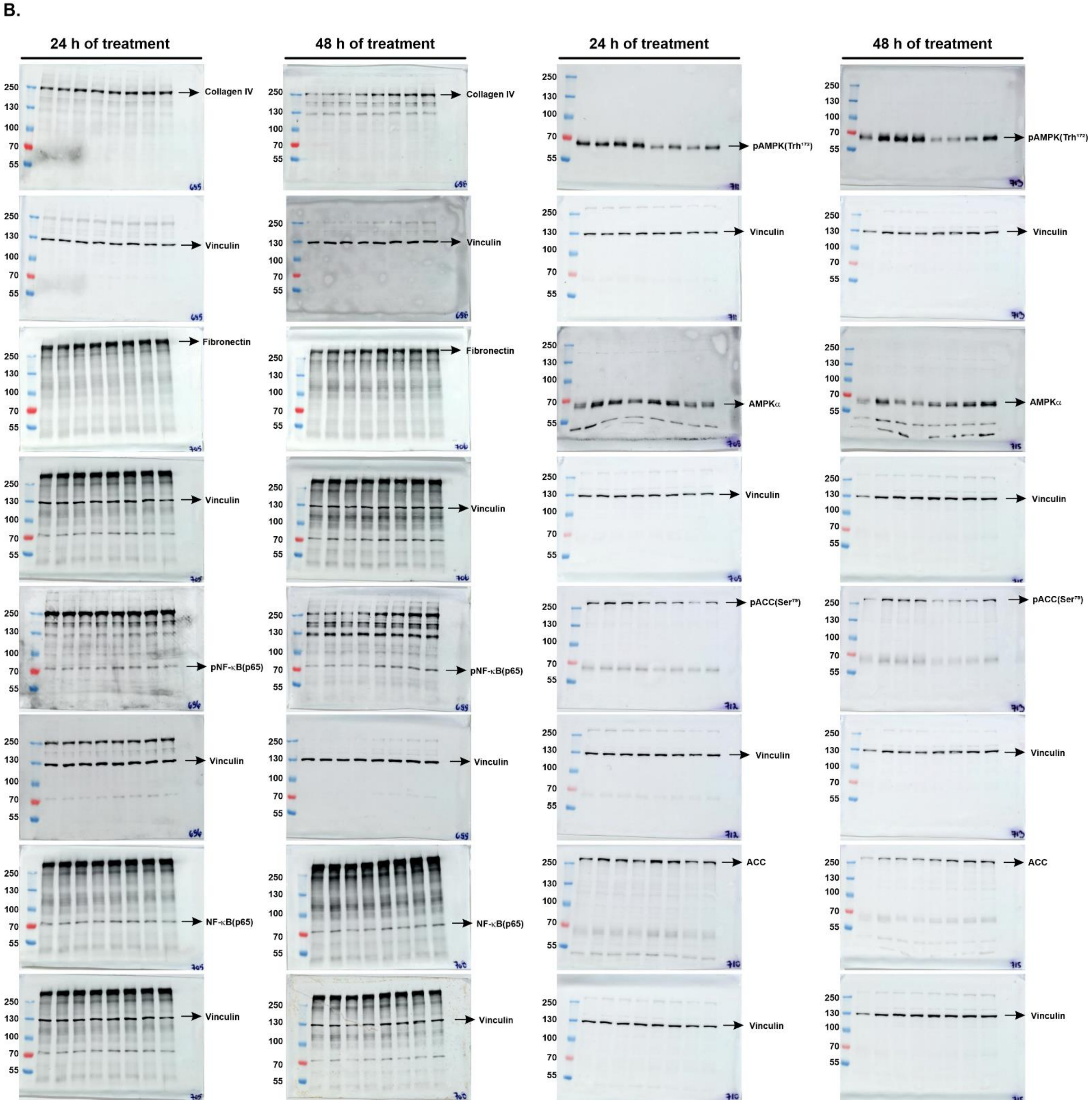

There are the full-length gels and blots of type IV collagen, fibronectin, pNF-κB(p65), NF-κB(p65), pAMPK(Trh^{172}), AMPKα, pACC(Ser^{79}) and ACC, and its Vinculin, in Supplementary Figure 2B. In all the gels, from left to right side, the first blots represent the molecular markers, the second set of four blots represent the expression level in HK-2 cell exposed to NG for 24 hours or 48 hours, the third set of four blots represent the expression level in HK-2 cell exposed to HG for 24 hours or 48 hours.
